# Effect of the Zn/Mg Ratio on Microstructures, Mechanical Properties and Corrosion Performances of Al-Zn-Mg Alloys

**DOI:** 10.3390/ma13153299

**Published:** 2020-07-24

**Authors:** Keda Jiang, Yanquan Lan, Qinglin Pan, Yunlai Deng

**Affiliations:** 1 Light Alloy Research Institute, Central South University, Changsha 410083, China; kodajiang@126.com (K.J.); pql1964@126.com (Q.P.); 2 School of Materials Science and Engineering, Central South University, Changsha 410083, China; luckdeng.msea@gmail.com

**Keywords:** Zn/Mg ratio, Al-Zn-Mg alloys, microstructures, mechanical properties, corrosion resistance

## Abstract

The effect of the Zn/Mg ratio on microstructures, mechanical properties and corrosion performances of Al-Zn-Mg alloys was studied. Microstructures were characterized using the optical microscope (OM), scanning electron microscope (SEM) and transmission electron microscope (TEM). Tensile tests, intergranular corrosion (IGC) and stress corrosion cracking (SCC) tests were conducted to study the properties. Microstructures results indicated that with the decrease of the Zn/Mg ratio, the recrystallization proportion and the fraction of second phase decreased, while the size of η’ (MgZn_2_) phases in grain interior also significantly decreased. The number density of η’ phases in grain interior increased and grain boundary precipitates developed discontinuous distribution with the decrease of the Zn/Mg ratio. These microstructures contributed to the significant improvement of the strength and corrosion resistance. The tensile strength and yield strength increased by 34.1% and 47.4%, respectively, with the Zn/Mg ratio decreased from 11.4 to 6.1. Calculating results indicated that the enhancement of strength mainly contributed from the solid-solution strengthening, grain-boundary strengthening and precipitation strengthening. The intergranular corrosion degree was greatly relieved and the stress corrosion sensitivity index decreased from 0.031 to 0.007 with the Zn/Mg ratio decreased from 11.4 to 6.1.

## 1. Introduction

Al-Zn-Mg alloys have been widely used in vehicles, buildings, bridges and other fields as light material because of their lower density, higher strength, better weldability and formability [1,2,3]. It is well known [4] that this alloy is an age-hardening alloy and its precipitation sequence is: the supersaturated solid solution (SSS) → the segregation zone (GP zone) → transition phases η’ (metastable phases MgZn_2_) → equilibrium phases η (MgZn_2_). Different aging processes can control the morphology of precipitates. Semi-coherent η’ precipitates with high density formed in grain interior under the peak aging (T6) condition, which would improve mechanical properties of Al-Zn-Mg alloys. However, continuous distribution of grain boundary precipitates leads to poor corrosion resistance [5]. Corrosion resistance is an important property in 7xxx series aluminum alloys and poor corrosion resistance limits the application of this alloy.

In order to improve the corrosion resistance of 7xxx series aluminum alloys, many researches have been carried out by regulating heat treatment processes and optimizing the composition of alloys. The RRA (retrogression and re-aging) process can regulate the grain boundary precipitates to discontinuous distribution, thus improve the corrosion resistance [6,7]. During the RRA process, η’ phases in grain interior get coarsened and partly transformed to the non-coherent η phases, so the number density of η’ precipitates gets reduced. Less η’ precipitates weaken the precipitation strengthening, thus the strength of RRA treated Al-Zn-Mg alloy is lower than the T6-aged alloy [8,9]. Zn and Mg are two main elements in 7xxx alloys. Increasing the total addition of Zn and Mg elements will contribute to the increase of the volume fraction of precipitates, such as MgZn_2_ phases in grain. However, the toughness and stress corrosion resistance of Al-Zn-Mg alloys get worse [10,11]. The effect of the Zn/Mg ratio on the strength is due to the change of precipitation sequence during the aging process. When the Zn/Mg ratio is higher than 2.2, GP zones mainly transformed into η’ phases. When the Zn/Mg ratio is lower than 2.2, GP zones mainly transformed into T’ or T phases [Mg_32_(Al, Zn)_49_] [12,13]. It was also found that the Zn/Mg ratio influenced the quenching sensitivity of Al-Zn-Mg alloys [14]. When the alloy was slowly quenched in air, the quenching sensitivity of the alloy with a lower Zn/Mg ratio was 7–11%, which is higher than that of the alloy with a higher Zn/Mg ratio. Researches on the strength and quench sensitivity of alloys with different Zn/Mg ratios were more comprehensive, while the research on the corrosion resistance of alloys with different Zn/Mg ratios was rare.

In this study, the effect of Zn/Mg ratios on mechanical properties and corrosion properties was studied in Al-Zn-Mg alloys with the same total amount of Zn and Mg additions. The relationship of microstructures and properties is built to provide a reference for the composition optimization and performance control of the Al-Zn-Mg extrusion profiles.

## 2. Experimental

The material used in this study was Al-Zn-Mg extruded profiles with a thickness of 4.5 mm, which were bought as commercial materials from the Yanconla company, Zouchen, China. The total amount of Zn and Mg additions of the alloys was about 6.6 wt% and the Zn/Mg ratio was set as 11.4 (marked as 1# alloy), 8.1 (marked as 2# alloy) and 6.1 (marked as 3# alloy), respectively. The specific chemical composition of three alloys is shown in Table 1. The extruded samples were solution treated at 480 °C for 1 h, then quenched into cold water within 3 s. Subsequently, the alloys were subjected to single stage artificial aging at 120 °C for 24 h.

**Figure 1:**
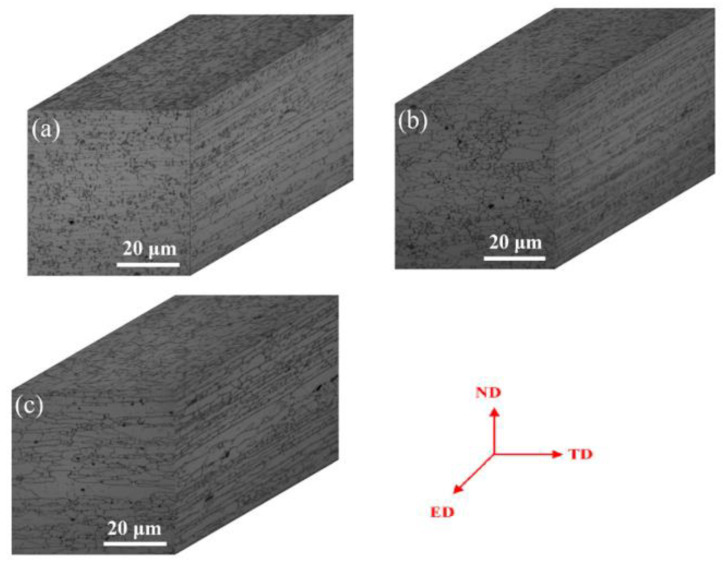
Three-dimensional view of microstructures of alloys with different Zn/Mg ratios: (**a**) 1# alloy; (**b**) 2# alloy; (**c**) 3# alloy.

The metallographic observation was conducted using the Optical Microscope (OM) (Olympus, New York, NY, USA) and samples were prepared through a conventional mechanical polishing and then etched using the Graff reagent (3 g CrO_3_, 16 mL HNO_3_, 1 mL HF and 83 mL H_2_O) (selfmade, Changsha, China) to acquire the size and shape of grains. To quantify the recrystallized structures, samples were sectioned and polished normal to the extrusion direction, which is marked as ED. The normal direction of the extrusion is marked as ND, while the transverse direction is marked as TD, as shown in Figure 1 and then examined using the electron backscattered diffraction (EBSD) technique using the SEM (Zeiss EVO MA10) (Zeiss, Jena, Germany). EBSD data were analyzed by Chanel 5 software (Zeiss, Jena, Germany) to obtain the grain orientation, grain boundary angle distribution and recrystallization fraction of samples. The morphology of second phase in alloys was observed using the SEM and compositions of second phase was tested using the energy dispersive X-ray spectroscopy (EDS) (Zeiss, Jena, Germany). Precipitates were characterized by the transmission electron microscope (TECNAIG2 F20) (FEI, Hillsboro, America) operated at 200 kV. Thin foils were preground to 100 μm, then twin-jet electro polished in solution with 20 vol% HNO_3_ (Shenghua Technology, Changsha, China) and 80 vol% CH_3_OH at −25 ℃ (Shenghua Technology, Changsha, China). Image J software (NIH Image, New York, NY, USA) was used to measure the amount of second phase and precipitates. Five SEM images with 200× magnification were used to measure the amount of second phases and five TEM images with 100,000× magnification were used to measure the number density and size of precipitates.

Mechanical properties tests were conducted on tensile specimens by the DDL 100 machine (Changchun Institute of Machinery, Changchun, China) at room temperature with a tensile speed of 2 mm/min. The parallel length and width of tensile specimens were 25 mm and 6 mm, respectively.

The intergranular corrosion (IGC) test was performed according to the IGC method described in ref [15]. Samples were submerged in premade solution (57 g NaCl, 10 mL H_2_O_2_, diluted to 1 L water) (Shenghua Technology, Changsha, China) at 35 ℃ for 6 h after being degreased using the ethanol and sealed using the rosin except the ND–TD plane. The susceptibility of SCC was evaluated using the slow strain rate test (SSRT) with a strain rate of 10^−6^ s^−1^. Specimens were tested by the RSW50 machine (Changchun Institute of Machinery, Changchun, China) and the test direction was along the extrusion direction. Specimens were tested in 3.5 wt% NaCl solution (Shenghua Technology, Changsha, China) at 25 ℃ and tests were also conducted in air for comparison. The susceptibility of SCC was calculated by the ratio of *I_SSRT_.* The expression was defined as follows [16]:

*I_SSRT_* = 1 − [R_m (NaCl)_ × (1 + δ _(NaCl)_)]/[R_m (Air)_ × (1 + δ _(Air)_)] (1)

where *R_m_* is the tensile strength, *δ* is the elongation. The SCC susceptibility would increase when the parameter *I_SSRT_* increased from 0 to 1.

## 3. Results and Discussions

### 3.1. Effect of the Zn/Mg Ratios on Microstructures

Three-dimensional views of granular microstructures of Al-Zn-Mg alloys with different Zn/Mg ratio are shown in Figure 1. The top surface layer (ED–TD) of the alloy showed heterogeneous distribution of coarse grains. Grains of cross section and longitudinal section performed elongated fiber structure and the aspect ratio of longitudinal section grains is much larger than that of cross section grains. It can be seen from Figure 1 that there is no significant difference in the grain size with the decrease of the Zn/Mg ratio from 11.4 to 6.1.

**Figure 2:**
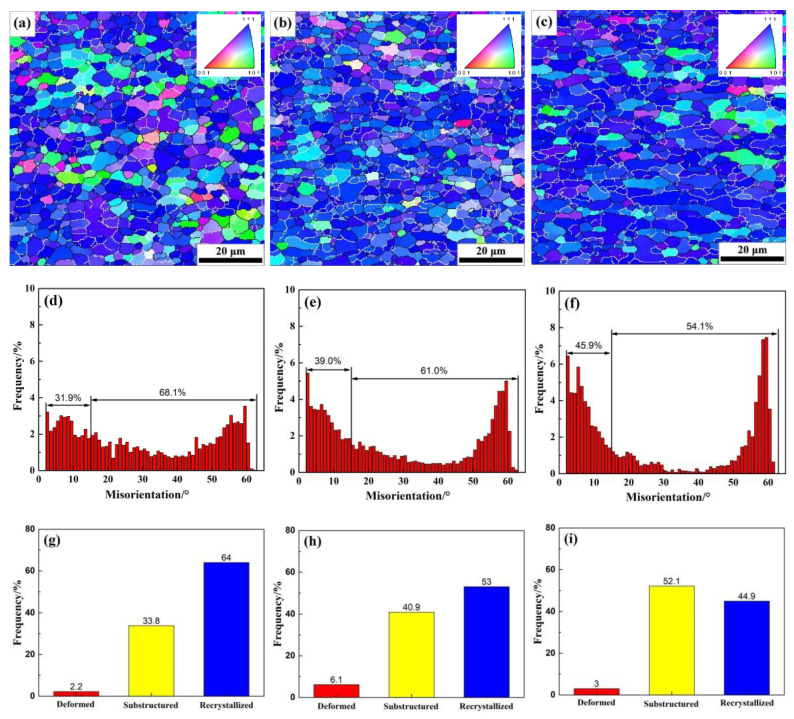
Electron backscattered diffraction (EBSD)maps (top surface layer (ED–TD) plane) of grain orientation and distribution of grain boundary angles and recrystallization microstructures of alloys with different Zn/Mg ratios (**a**,**d**,**g**) 1# alloy; (**b**,**e**,**h**) 2# alloy; (**c**,**f**,**i**) 3# alloy.

EBSD maps from cross sections (ND–TD) of alloys with different Zn/Mg ratio are shown in Figure 2. The distribution of grain orientation, distribution of grain boundary angle and recrystallization microstructures are also presented in Figure 2. In Figure 2a–c, the white solid line represents the small angle grain boundary (2° < θ < 15°) and the black solid line represents the large-angle grain boundary (θ > 15°) and the average grain size of the alloys with different Zn/Mg ratio is about 3–4 μm. There was no significant effect on the grain size of alloys with different Zn/Mg ratios. The distribution of corresponding angular in grain boundary is shown in Figure 2d–f. When the Zn/Mg ratio was 11.4, 8.1 and 6.1, the corresponding large-angle grain boundary ratio would be 68.1%, 61.0% and 54.1%, respectively. The proportion of grain boundary with large angle gradually decreased with the decrease of the Zn/Mg ratio. However, the misorientation distribution of the grain boundary angle had a distinguished behavior. The frequency of low- and high-angle grain boundaries increased as the Zn/Mg ratio decreased, especially the angles between 30–50° decreased significantly. The alloy with a Zn/Mg ratio of 6.1, showed the lowest number of grains randomly distributed due to the insufficient recrystallization occurred in this alloy. Results in Figure 2g–i indicate that when the Zn/Mg ratio was 11.4, 8.1 and 6.1, the proportion of recrystallization fraction would be 64.0%, 53.0% and 44.9%, respectively. With the decrease of the Zn/Mg ratio, the proportion of the recrystallization structure in the alloy gradually decreased. Therefore, as the Mg content in the alloy increased, the number of substructure grains retained after being solution heat-treated at 480 °C became more numerous. Thus, the work hardening strength will be more effective.

**Figure 3:**
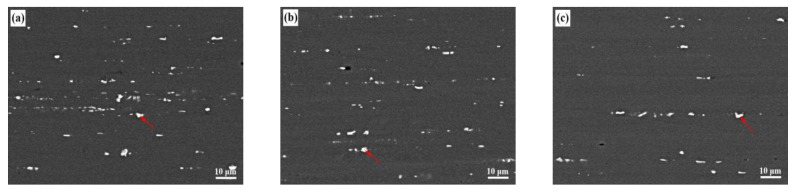

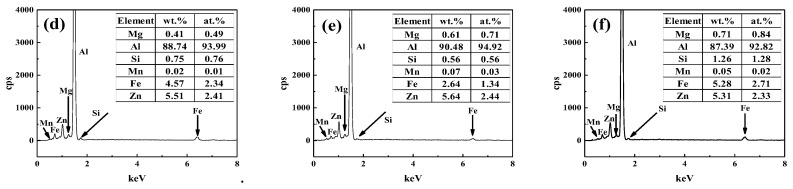
SEM images (ED–TD plane) and EDS results of alloys with different Zn/Mg ratios; (**a**,**d**) 11.4; (**b**,**e**) 8.1; (**c**,**f**) 6.1.

SEM images and EDS results of alloys with different Zn/Mg ratio are shown in Figure 3. White irregular coarse second phases were distributed unevenly along the extrusion direction. EDS results indicated that white coarse second phases are mainly the AlFeMnSi phase, while small amount of Mg element and large amount of Zn element dissolved into the matrix. The amount of second phases in multiple fields of view were calculate by the Image J software. When the Zn/Mg ratio was 11.4, 8.1 and 6.1, the number density of the coarse second phases would be 9609 mm^−2^, 7852 mm^−2^ and 5216 mm^−2^, respectively. AlFeMnSi phases also contained Zn elements, as the EDS results in Figure 3d–f showed. With less Zn content in the matrix, there would be less coarse second phases. Results in Figure 3a–c indicated that the amount of noncoarse particles decreased with the increase of Mg content. Therefore, the fraction of the coarse second phases significantly decreased with the decrease of the Zn/Mg ratio.

**Figure 4:**
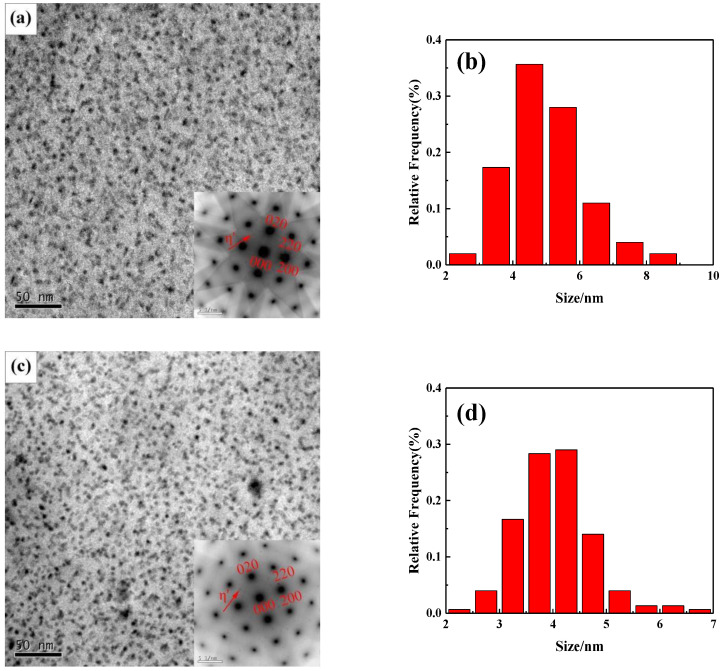

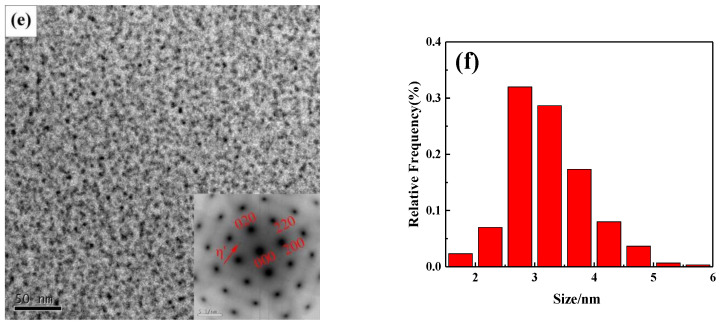
TEM images (ED–TD plane) and size distribution histogram of precipitates in alloys with different Zn/Mg ratios: (**a**,**d**) 1# alloy; (**b**,**e**) 2# alloy; (**c**,**f**) 3# alloy.

Figure 4a,c,e show the TEM images of precipitated phases in alloys with different Zn/Mg ratios and the electron incident direction was < 100 > _Al_. After the T6 heat treatment, short rod-shaped second phase particles precipitated uniformly in grain interior. It can be estimated as η’ phase from the selected area electron diffraction (SED) map. The number density of precipitates was counted by Image J software. Results showed that the number density of η’ phases in the grain interior significantly increased with the decrease of the Zn/Mg ratio. For the Zn/Mg ratio was 11.4, 8.1 and 6.1, the number density of η’ phases is 66,521 μm^−2^, 87,863 μm^−2^ and 118,142 μm^−2^, respectively. The size of η’ phases was measured and proportions of size was obtained through the normal distribution calculation method. As shown in Figure 4b,d,f, when the Zn/Mg ratio is 11.4, the size of η’ phases was 4–6 nm. When the Zn/Mg is 8.1, the size of η’ phases was 3.5–4.5 nm. When the Zn/Mg ratio was 6.1, the size of η’ phased was 2.5–3.9 nm. Results indicated that the size of η’ phases significantly decreased with the decrease of the Zn/Mg ratio.

**Figure 5:**
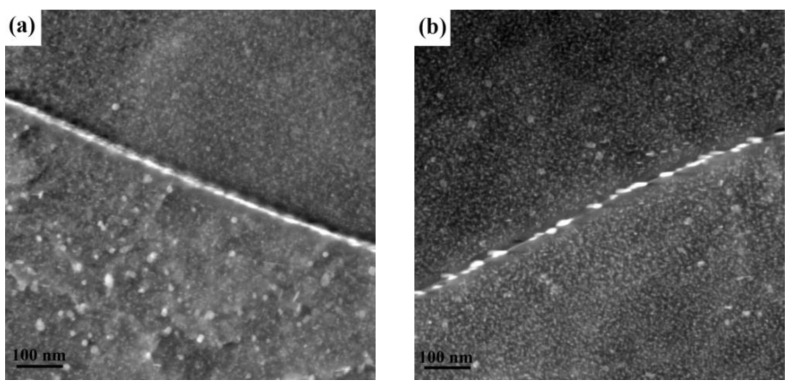

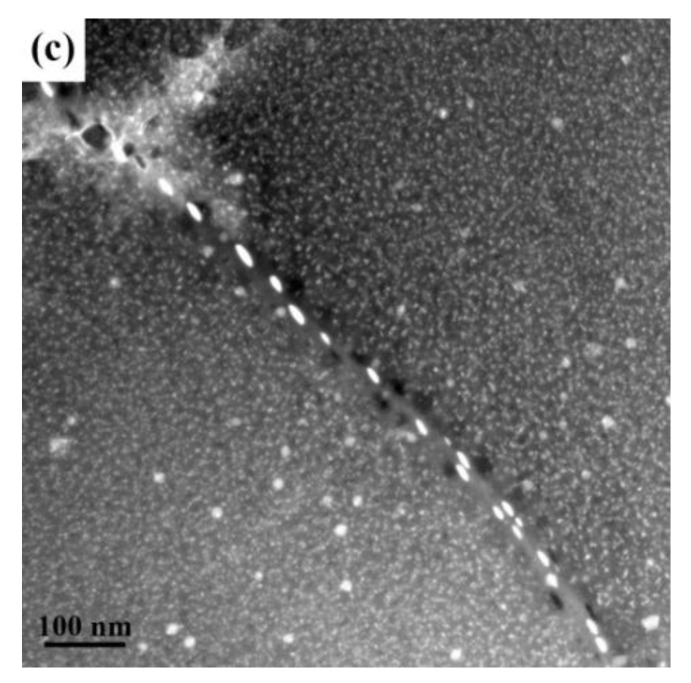
STEM images (ED–TD plane) of precipitated phase at grain boundaries of alloys with different Zn/Mg ratios: (**a**) 1# alloy; (**b**) 2# alloy; (**c**) 3# alloy.

STEM (Scanning transmission electron microscope) images of the grain boundary precipitates (GBP_S_) in alloys with different Zn/Mg ratios are shown in Figure 5. The white and thick η phases precipitated on the grain boundary. In Figure 5, the width of precipitates free zone (PFZ) around grain boundaries was not obviously different for different Zn/Mg ratios. However, the GBPs became coarse with the decrease of the Zn/Mg ratio. When the Zn/Mg ratio was 11.4, the GBP_S_ were dense and continuously distributed. The GBPs became discontinuous distribution when the Zn/Mg ratio decreased to 8.1. When the Zn/Mg ratio was further decreased to 6.1, the GBP_S_ became interrupted distributed and the gap between GBP_S_ became larger. Results indicated that with the decrease of the Zn/Mg ratio, the gap between the GBP_S_ gradually became larger and the continuous distribution state changed to discontinuous distribution.

### 3.2. Effect of the Zn/Mg Ratios on Mechanical Properties

**Figure 6:**
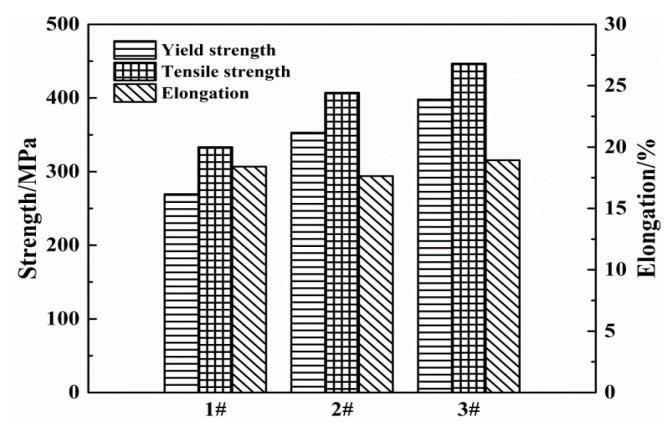
Mechanical properties (ED direction on ED–TD plane) of alloys with different Zn/Mg ratios.

Figure 6 shows results of room temperature tensile tests of alloys with different Zn/Mg ratios. The tensile strength of three alloy was 333.2 MPa, 407.3 MPa and 446.9 MPa for alloys with the Zn/Mg ratio was 11.4, 8.1 and 6.1, respectively. The yield strength was 269.4 MPa, 352.9 MPa and 397.2 MPa for alloys with the Zn/Mg ratio was 11.4, 8.1 and 6.1, respectively. The tensile strength and yield strength increase by 34.1% and 47.4%, respectively, with the Zn/Mg ratio decreased from 11.4 to 6.1. The elongation of alloys with the Zn/Mg ratio of 11.4, 8.1 and 6.1 was 18.4%, 17.6% and 18.9%, respectively.

The strengthening mechanisms of age hardenable Al-Zn-Mg alloy are solid-solution strengthening, grain-boundary strengthening, dislocation strengthening and precipitation strengthening. These strengthening mechanisms are related to the microstructure of alloys. Zn and Mg are two main alloying elements in the Al-Zn-Mg alloy and the ratio between them significantly affects the microstructure of the alloy through the precipitation during the aging process. The strength model can be used to calculate the contribution of microstructures to strength [17], as follows:

(2) $$\sigma_{y}=\Delta \sigma_{gb}+M(\Delta \tau_{0}+\Delta \tau_{ss}+\Delta \tau_{d\&ppt})$$

where $\Delta \sigma_{gb}$ is the grain-boundary strengthening contribution [18], depending on the average grain sizes, which are 3.9 μm, 3.2 μm and 3.7 μm for alloys with the Zn/Mg ratio is 11.4, 8.1 and 6.1, respectively. *M* is the average Taylor factor which depending on the components and volume fractions of the textures, which are 3.2, 3.1 and 3.5 for three alloys. $\tau_{0}$ is the intrinsic critical resolved shear stress of pure Al, set as 10 MPa. $\Delta \tau_{ss}$ is the solid-solution strengthening contribution, depending on the types and concentrations of solutes. $\Delta \tau_{d\&ppt}$ is the dislocation and precipitation strengthening contribution, depending on the dislocation density and the type, size and number density of precipitates.

(3) $$\Delta \sigma_{gb}=\alpha_{2}Gb\left[\left(1-f_{ReX}\right)\left(\frac{1}{\delta }\right)+f_{ReX}\left(\frac{1}{D}\right)\right]$$

where *G* is the shear modulus of Al, which is 28 GPa. *b* is the Burgers vector, which is 0.286 nm. $f_{ReX}$ is the recrystallized volume fraction, *δ* is the (sub-)grain size or cell size in the unrecrystallized part of the alloy, $\alpha_{2}$ is a constant (typically equal to 2 [19]) and *D* is the grain size of recrystallized microstructures. The calculating result of grain-boundary strengthening are shown in Table 2. With the Zn/Mg ratio decreased from 11.6 to 6.1, the strengthening from grain boundaries increased from 4.9 MPa to 13 MPa.

We assumed that the contribution from each element is additive, the solution hardening potential ($\Delta \tau_{ss}$ ) can be calculated according to Equation (5) [20] and the results are shown in Table 3.

(4) $$\Delta \tau_{ss}=\sum k_{j}c_{j}^{n_{ss}}$$

where *k* is a constant related to properties of the related solute *j*, *c* is the solute concentration. The corresponding strengthening coefficients of Zn, Mg and Cu elements are 3.085, 20.081 and 12.431, respectively. $n_{ss}$ is taken as 2/3. As the strengthening coefficient of the Mg element is higher than the Zn element, the solid-solution strengthening increased with the decrease of the Zn/Mg ratio.

The precipitation strengthening was mainly due to the hindrance from precipitates to the dislocation movement during the deformation. The decrease of Zn/Mg ratio changed the morphology of precipitates, as shown in Figure 4. The precipitation strengthening can be described according to the Orowan equation [21], as follows:

(5) $$\Delta \tau_{d\&ppt}=2\left(\frac{Gb}{4\pi \sqrt{1-v}}\right)\left(\frac{1}{\lambda }\right)(ln\frac{d}{r_{0}})$$

(6) $$\lambda =\left(0.538\sqrt{\frac{2\pi }{3f}}-\frac{\pi }{4}\right)d$$

where *G* is the shear modulus of Al, *b* is the Burgers vector, *v* is the Poisson’s ratio, *λ* is the effective interparticle spacing of coarse second phases, *d* is the average diameter of precipitates and $r_{0}$ is the core radius of dislocations, *f* is the volume fraction of precipitates. Assuming that the precipitate is spherical, then *f* is obtained according to the following formula:

(7) $$f=N_{v}V=N_{v}\pi \;{\left(d\right)}^{3}/6$$

The number density of precipitates in Al-Zn-Mg alloys increased with the decrease of the Zn/Mg ratio, which is mainly affected by the content of Zn and Mg elements in the alloy. The mass fraction ratio of three alloys are 11.4, 8.1 and 6.1, respectively, which are higher than the mass ratio of MgZn_2_ phase (Zn/Mg = 5.4). It indicated that the excessive of Zn content and the number density of MgZn_2_ phase mainly depends on the content of the Mg element. The number density of precipitates was obviously reduced in alloys with a higher Zn/Mg ratio (11.4 and 8.1) due to lower Mg content compared with the alloy with a lower Zn/Mg ratio (6.1). Meanwhile, the remain Zn content in the alloy would form impurity phases, such as AlFeMnSi phases (as shown in Figure 3. The number density of AlFeMnSi phase obviously decreased with the decrease of the Zn/Mg ratio. Therefore, the difference of the number density of precipitates in alloys with different Zn/Mg ratios is mainly caused from the difference of the Mg content.

The main strengthening mechanism of Al-Zn-Mg alloys is the precipitation strengthening and the metastable η’ phase is mainly precipitated in the peak aging state (T6) [17]. The strengthening mechanism is that the dislocation line bypass through η’ phases, which increase the resistance of dislocation movement. With the decrease of Zn/Mg ratio, the number density of η’ phases increases, thus the resistance of dislocation movement increases. The calculating result of precipitation strengthening was listed in Table 4.

The calculating yield strength were 294.4 MPa, 318.3 MPa and 394.1 MPa for alloys with the Zn/Mg ratio is 11.4, 8.1 and 6.1, respectively. Therefore, the strength of alloy with a lower Zn/Mg ratio is higher than the alloy with a higher Zn/Mg ratio, which is corresponding to tensile test results. Experimental yield strength of alloy with the Zn/Mg ratio is 8.1 performed higher strength than theoretical, while on alloys with the Zn/Mg ratio is 11.4 and 6.1 occurred the opposite. This difference mainly causes from ignoring the strength increment from coarse second phases. As alloys with the Zn/Mg ratio is 11.4 and 8.1 presented higher number density of the coarse second phases with 9609 mm^−2^ and 7852 mm^−2^, respectively, compared with 5216 mm^−2^ of the alloy with the Zn/Mg ratio equal to 6.1.

### 3.3. Effect of Zn/Mg Ratios on Corrosion Performances

**Figure 7:**
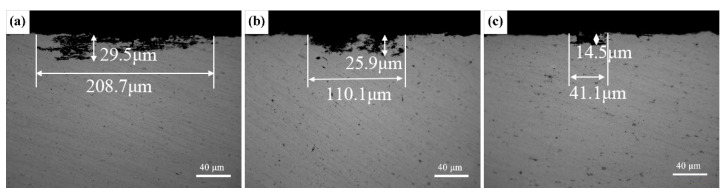
Intergranular corrosion morphologies (ED–TD plane) of alloys with different Zn/Mg ratios: (**a**) 1# alloy; (**b**) 2# alloy; (**c**) 3# alloy.

Figure 7 shows metallographic cross-sections (ND–TD) with the maximum corrosion depth and width of three different Zn/Mg ratio alloys after the intergranular corrosion. The corrosion attack would like to develop along grain boundaries. Obviously, 1# alloy was the most susceptible to the corrosion attack. Clear network microstructure could be seen and the maximum corrosion depth and width extended up to 29.5 μm and 208.7 μm, respectively. The corrosion sensitivity of 2# alloy was lower with the maximum depth and width were 25.9 μm and 110.1 μm, respectively. For 3# alloy, the maximum depth and width were 14.5 μm and 41.1 μm, respectively, suggesting that the corrosion degree was greatly relieved with the decrease of the Zn/Mg ratio.

**Figure 8:**
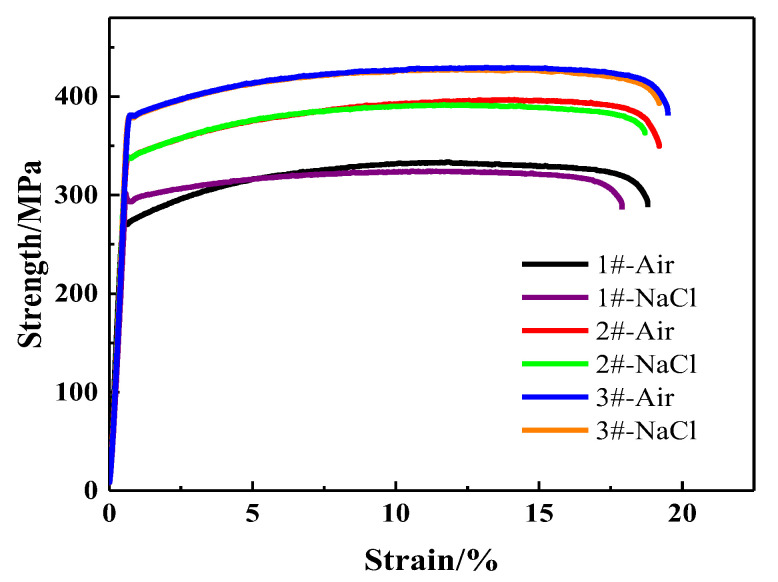
Slow strain rate testing (SSRT) curves for three alloys (ED direction on ED–TD plane): (**a**) in air; (**b**) in 3.5 wt% NaCl solution.

Figure 8 shows the stress-strain curves obtained by slow strain rate tests of alloys with different Zn/Mg ratios in air and in the 3.5 wt% NaCl solution. The corrosion environment will lead to the decrease of the elongation and the extent of the decrease is various with different Zn/Mg ratios. As shown in Figure 8, the elongation of the alloy with the Zn/Mg ratio is 11.4 was significantly lower in NaCl solution than in the air. When the Zn/Mg ratio was reduced to 8.1 or 6.1, the elongation in air and NaCl solution environment has less difference. The slow strain rate results are shown in Table 5. When the Zn/Mg ratio was 11.4, the tensile strength in NaCl solution decreased by 8.0 MPa compared with that in air and the elongation decreased by 5.0%. When the Zn/Mg ratio was 8.1, the tensile strength decreased by 4.5 MPa and the elongation decreased by 2.6%. When the Zn/Mg ratio was 6.1, the tensile strength decreased by 2.1 MPa and the elongation decreased by 1.5%. When the Zn/Mg ratio is 11.4, 8.1 and 6.1, the stress corrosion sensitivity index is 0.031, 0.015 and 0.007, respectively. The stress corrosion resistance of Al-Zn-Mg alloys was improved with the decrease of the Zn/Mg ratio.

The intergranular corrosion happened in the alloy due to the potential of MgZn_2_ phases on the grain boundary is lower than the matrix, thus anodic dissolution occurs preferentially. When the GBPs is continuously distributed, a coherent corrosion channel is formed, which is manifested in the corrosion form along the grain boundary. Therefore, intergranular corrosion is mainly related to the distribution of GBPs and continuous distribution will lead to rapid expansion of corrosion. As the STEM images in Figure 5 show, with the decrease of the Zn/Mg ratio, GBPs gradually changed from continuous distribution to intermittent distribution. The gap between GBPs gradually became larger to prevent corrosion expand along the grain boundary. In addition, EBSD analysis (Figure 2) showed that the recrystallization fraction of the alloy decreased with the decrease of the Zn/Mg ratio, which was related to the decrease of the proportion of large-angle grain boundaries [22]. However, large-angle grain boundaries of recrystallized grains were easy to form active corrosion zone, causing intergranular corrosion in a certain recrystallization stage [23]. Therefore, under the joint action of large-angle grain boundaries and GBPs, the alloy with a higher Zn/Mg ratio was more easily affected by the intergranular corrosion than the alloy with a lower Zn/Mg ratio. It indicated that the reduction of the Zn/Mg ratio is conducive to improve the intergranular corrosion property of Al-Zn-Mg alloys.

The SCC sensitivity of alloys decreased with the decrease of the Zn/Mg ratio. According to the perspective of anodic dissolution theory, it is mainly due to the distribution state of GBPs changes from continuous to discontinuous. The alloy with a higher Zn/Mg ratio forms less effective MgZn_2_ phase for a low content of Mg, so the remaining Zn content is relatively more. Moreover, the Zn content can increase the potential difference between GBPs and the grain interior [24], which increasing the tendency of the stress corrosion. According to the theory of hydrogen embrittlement, the hydrogen produced in the corrosion environment is more likely to migrate to the vicinity of the GBPs through dislocations. Hence, the bonding strength of GBPs reduced and the grain boundary became prone to cracking. Therefore, the SCC sensitivity of the Al-Zn-Mg alloy with a higher Zn/Mg ratio is higher than that of the alloy with a lower Zn/Mg ratio.

## 4. Conclusions

The effect of the Zn/Mg ratio on microstructures, mechanical properties and corrosion resistance of Al-Zn-Mg extruded profiles were studied.

1. With the decrease of the Zn/Mg ratio, the recrystallization proportion decreased, the number fraction of the second phase decreased and the size of the η’ phase in grain interior significantly decreased. While the number density of η’ phase in grain interior increased and the gap between the grain boundary precipitates increased with the decrease of Zn/Mg ratio.
2. The tensile strength and yield strength are increased by 34.1% and 47.4%, respectively, with the Zn/Mg ratio decreased from 11.4 to 6.1. The elongation of alloys with the Zn/Mg ratio of 11.4, 8.1 and 6.1 is 18.4%, 17.6% and 18.9%, respectively. Experimental yield strength of alloy with the Zn/Mg ratio of 8.1 was higher than theoretical, while on alloys with the Zn/Mg ratio of 11.4 and 6.1 occurred the opposite. Calculating results indicated that the enhancement of strength mainly contributed from the solid-solution strengthening, grain-boundary strengthening and precipitation strengthening.
3. The intergranular corrosion degree is greatly relieved with the decrease of the Zn/Mg ratio. The stress corrosion sensitivity index is 0.031, 0.015 and 0.007 for the Zn/Mg ratio with 11.4, 8.1 and 6.1, respectively. The stress corrosion resistance of alloys improved with the decrease of the Zn/Mg ratio.

## Figures and Tables

**Table 1:** Chemical composition of Al-Zn-Mg alloys with different Zn/Mg ratios (wt%).

Alloys	Zn/Mg	Zn and Mg	Zn	Mg	Cu	Mn	Cr	Ti	Zr	Fe	Si	Al
1#	11.4	6.57	6.04	0.53	0.17	0.25	0.12	0.08	0.20	0.15	0.06	bal.
2#	8.1	6.57	5.85	0.72	0.16	0.23	0.15	0.07	0.17	0.15	0.06	bal.
3#	6.1	6.61	5.68	0.93	0.16	0.26	0.14	0.08	0.19	0.14	0.07	bal.

**Table 2:** Calculating strength from grain boundaries strengthening.

Zn/Mg Ratios	*δ* (μm)	*D* (μm)	$f_{ReX}$ (%)	$\Delta \sigma_{gb}$ (MPa)
11.4	2.5	3.9	64	4.9
8.1	1.2	3.2	53	8.9
6.1	0.8	3.7	44.9	13.0

**Table 3:** Solute concentration of samples in matrix (wt%) and calculating results of solid-solution strengthening.

Zn/Mg Ratios	Zn	Mg	Cu	$\Delta \tau_{ss}$ (MPa)
11.4	6.04	0.53	0.17	27.2
8.1	5.85	0.72	0.16	29.8
6.1	5.68	0.93	0.16	32.6

**Table 4:** Statistical results of precipitation strengthening.

Zn/Mg Ratios	*d* (nm)	*N_v_* (μm^3^)	$\Delta \tau_{p}$ (MPa)
11.4	4.3	6.65 × 10^4^	68.56
8.1	4.0	8.79 × 10^4^	74.12
6.1	3.7	1.18 × 10^5^	80.30

**Table 5:** Slow strain rate test (SSRT) results (ED direction on ED–TD plane) of alloys with different Zn/Mg ratios.

Alloy	Corrosive Environment	*Rm* (MPa)	Elongation (%)	*I_SSRT_*
1#	Air	332.58	18.8	0.031
	3.5 wt% NaCl	324.61	17.9	
2#	Air	395.73	19.2	0.015
	3.5 wt% NaCl	391.22	18.7	
3#	Air	429.67	19.5	0.007
	3.5 wt% NaCl	427.57	19.2	
